# Quantitative and qualitative analysis of three DNA extraction methods from soybean, maize, and canola oils and investigation of the presence of genetically modified organisms (GMOs)

**DOI:** 10.1016/j.fochms.2024.100201

**Published:** 2024-03-21

**Authors:** Melika Vahdani, Mohammad Ali Sahari, Mehrnaz Tanavar

**Affiliations:** a Department of Food Science and Technology, College of Agriculture, Tarbiat Modares University, P. O. Box, 14115-336 Tehran, Iran; b Department of Plant Genetics and Breeding, College of Agriculture, Tarbiat Modares University, Tehran, Iran

**Keywords:** CTAB (PubChem CID: 5974), Hexane (PubChem CID: 8058), Chloroform (PubChem CID: 6212), Isopropanol (PubChem CID: 3776), Ethanol (PubChem CID: 702), Methanol (PubChem CID: 887), Acetic acid glacial (PubChem CID: 176), Coomassie brilliant blue G-250 (PubChem CID: 6324599), Genetically modified organisms (GMOs), Polymerase chain reaction (PCR), DNA extraction, PCR inhibitors, Crude and refined edible oils

## Abstract

The objective of this study was to develop a DNA-based method for the identification and tracking of edible oils, which is important for health management. Three different DNA extraction methods (CTAB, MBST kit, and manual hexane-based method) were used to obtain high-purity DNA from crude and refined soybean, maize, and canola oils. PCR was then conducted using specific primers to identify the presence of genes related to each oil type and to assess transgenicity. The results showed that DNA was present in crude and refined oils, but in very low amounts. However, using method 3 for DNA extraction provided sufficient quantity and quality of DNA for successful PCR amplification. The study concluded that the main challenge in DNA extraction from oils is the presence of PCR inhibitors, which can be overcome using the manual hexane**-**based method. Also, the examination of protein presence in the oils using SDS-PAGE did not indicate any protein bands.

## Highlights

• To develop a DNA-based method for detection of edible oils;
• To enhance the quality and safety of oil products (soybean, maize and canola);
• Three different DNA extraction methods were used;
• CTAB, MBST kit, and manual hexane-based method for detecting of GMOs were used;
• To obtain high-purity DNA from crude and refined three oils;
• The protein presence in the oils using SDS-PAGE was investigated.

## 1 Introduction

Genetically modified crops are akin to their natural counterparts, with the distinction that through genetic engineering, they have been differentiated and improved in one or more traits compared to their natural varieties. Genetically Modified Organisms (GMOs) have significantly evolved over the past three decades through advanced genetic engineering methods (Luna et al., 2013). The global area of biotechnological products has increased remarkably from 1.7 million hectares in 1996 to 190.4 million hectares in 2019 (approximately 112-fold increase). This trend makes biotechnological products the fastest-adopted technology in recent years. Major GM crops include soybean (48.2 % of the global biotechnological product area), corn (32 %), cottonseed (13.5 %), and canola (5.3 %) (ISAAA, 2019).^1^

Despite the widely recognized benefits of GMOs, a significant portion of the world, non-governmental organizations, and the general public express doubts or opposition for various reasons, including concerns about the impact of GMOs on human health, animals, and the environment, as well as economic effects associated with the cultivation and use of genetically modified products (Luna et al., 2013, Liu et al., 2016). One of the most significant drawbacks of genetically modified products is the potential introduction of allergens and toxins to humans, leading to allergic reactions (Iioh et al., 2018).

As genetically modified (GM) products become increasingly diverse and accessible in global markets, the ability to detect and identify the presence of genetically modified organisms or non-inheritable contaminants throughout the food chain is crucial. This is essential for both raw material producers and the food industry to comply with the growing demand for legally mandated labeling of GM products (Germini et al., 2004, Kamle et al., 2017). Meeting the increasing consumer demand for awareness and transparent information regarding the components used in food production and implementing rigorous controls for evaluating organic products is of great importance (Germini et al., 2004). Therefore, the creation and development of sensitive, reliable, and specific methods (Kamle et al., 2017) for the identification and detection of genetically modified events by governmental regulatory bodies, international trade organizations, and industries utilizing these products have become increasingly significant (Schmidth et al., 2008).

In general, detection methods for GM products can be divided into two main categories: DNA-based methods and protein-based methods (Xia et al., 2021). The application of a DNA-based method for the detection and quantification of GMOs and achieving successful results in DNA amplification methods depends on the efficiency of DNA extraction methods and, consequently, the quality and quantity of the extracted DNA. This is considered a critical aspect in the analysis of complex and highly processed food matrices (Gryson, 2010, Luna et al., 2013, Costa et al., 2010, Costa et al., 2010).

The purity of the extracted DNA can be evaluated by measuring the absorbance ratios at A_260_/A_280_ and A_260_/A_230_ using a spectrophotometer or a NanoDrop. The most common analytical methods developed for GMO detection are DNA-based, such as Polymerase Chain Reaction (PCR) which is recognized as the most effective tools for examining GM foods, given the stability of DNA molecules during food processing (Bogani et al., 2009, Jinxia et al., 2011, Datukishvili et al., 2015, Iioh et al., 2018). Protein-based methods are not suitable and reliable for highly processed foods (Jinxia et al., 2011, Bogani et al., 2009).

The most accurate and widely used detection methods in various laboratories are based on specific DNA using PCR methods (Sepahian et al., 2019). DNA-based methods, especially PCR and Real-time PCR, are internationally recognized and recommended for the analysis of processed and thermally treated foods (Cottenet et al., 2019). The advantages of DNA-based methods, such as PCR, include high sensitivity, high specificity, repeatability, high discriminatory power, rapid processing time, and cost-effectiveness, enabling detection in raw and highly processed food items (Xia et al., 2019, Di Pinto et al., 2007).

Various methods have been developed for extracting DNA from oil samples. The initial method used for DNA extraction from oil samples was based on cetyl trimethyl ammonium bromide (CTAB). This method was employed in many studies (Testolin et al., 2002; Busconi et al., 2003, Testolin and Lain, 2005, Consolandi et al., 2008, Martins-Lopes et al., 2008, Giménez et al., 2010), but the purity of the extracted DNA was not very high (Nikolic et al., 2014). Therefore, some researchers modified the CTAB method using hexane and chloroform to achieve higher DNA purity (Consolandi et al., 2008, Giménez et al., 2010). Although the modified CTAB method had a better impact on the purity of the extracted DNA, many researchers have utilized DNA extraction methods based on specific binding of DNA to silica membrane, such as the Nucleospin kit (Consolandi et al., 2008). Some studies have been conducted using magnetic separation-based kits like the Wizard Magnetic Purification System for food materials (Breton et al., 2004, Testolin and Lain, 2005, Consolandi et al., 2008).

Various factors, including the target sequence (Gryson, 2010), physical and chemical processes (Gryson et al., 2002, Gryson et al., 2004a, Gryson et al., 2004b) such as mechanical stress, high temperature, pH changes, enzymatic and nuclease activities, fermentation, oil extraction, purification processes, soaking, and storage, can impact the primary structure of DNA. This can lead to events such as genomic DNA fragmentation, hydrolysis and random degradation, oxidation, and deamination of DNA (Meyer, 1999, Gryson et al., 2002, Kharazmi et al., 2003, Gryson, 2010, Shayan et al., 2017). In turn, this reduces the sensitivity of analysis, affecting the detection limit and quantification, potentially altering the qualitative and quantitative results of GMO analysis and making the interpretation of PCR analysis challenging. Despite this, DNA amplification is still possible. The length and composition of the amplified fragment, along with the efficiency of the extraction method, can impact test results, posing challenges in distinguishing additional DNA in the sample and mitigating PCR inhibitors (Gryson, 2010).

Recently, it may be essential to determine the authenticity of edible oils and detect possible fraud through DNA-based methods. Therefore, the development of a DNA extraction method is the first step toward achieving molecular traceability and the detection of adulteration, genetic modification, and varietal origin. Hence, the objective of this study is to compare different methods for extracting DNA from soybean, corn, and canola oils, and to provide an economical, reproducible, effective, and reliable method for detecting genetically modified oils in terms of food authenticity issues. Another important aspect regarding genetically modified oils is the presence of transgene proteins (herbicide or pest-resistant protein). In this study, the existence of proteins in oils was investigated using the SDS-PAGE method.

## 2 Materials and methods

### 2.1 DNA extraction

The samples of oils used in this research included crude and refined soybean oil, crude and refined corn oil, and crude and refined canola oil, with the default that all oils were of transgenic origin. Soybean and canola oils were obtained from the margarine oil factory, and the corn oil sample was sourced from the Glucosan Qazvin oil factory. Genomic DNA was extracted from a small scale (100 mg) of leaves from soybean, corn, and canola plants (powdered using liquid nitrogen) using the CTAB method (Rogers et al., 1985). This genomic DNA from non-transgenic plant leaves was used as a positive control in the PCR reaction. Genomic DNA extraction from oil was performed using three different methods:

#### 2.1.1 CTAB method

Genomic DNA extraction from oil samples was conducted following the method outlined in the study by Costa et al., 2010, Costa et al., 2010.

#### 2.1.2 MBST kit

In this method, DNA extraction from oil samples was performed according to the procedure described in the research by Shayan and colleagues (Shayan et al., 2017). The kit used for this extraction was provided by the Molecular Biological System Transfer (MBST) research group based in Iran/Germany.

#### 2.1.3 The manual hexane-based method

In this approach, DNA extraction from oil samples, with some modifications, followed the method outlined in the study by Piarulli et al. (2019). The extraction steps for oil were as follows:

First, all oil samples were vigorously mixed for 5 min, and 1 ml of each sample was transferred to a 2 ml vial. Subsequently, 500 µl of hexane was added to the samples, and they were shaken for 5 min. Then, the samples were centrifuged for 30 min at 4 °C and 12000 rpm. At this stage, two phases were observed, including a solid phase at the bottom of the vial, referred to as the pellet phase, and a liquid portion. In this experiment, both phases were separated for DNA extraction, and extraction was performed from both segments.

The liquid portion was transferred to new 2 ml vials, and then 400 µl of lysis buffer (50 mM Tris, 1 mM EDTA, 0.5 % *v/v* Tween 20, pH 8) was added to both the liquid and the pellet phases. The samples were mixed, and proteinase *K* (10 μl of 10 mg/ml) was added to the samples, which were then kept at 60 °C for 1 h. Subsequently, the samples were centrifuged for 15 min at 4 °C and 12000 rpm, and the aqueous phase was transferred to new separate vials.

To all samples, 500 µl of chloroform was added, and the vials were gently shaken for 5 min at 20 °C and centrifuged at 12000 rpm for 5 min. At this stage, two phases were formed; the upper phase, i.e., the aqueous phase containing DNA, was transferred to a new vial. Then, gently mixing with 500 µl of cold isopropanol, the samples were kept at −80 °C for 30 min and again centrifuged for 30 min at 4 °C and 14000 rpm. DNA sedimentation in the bottom of the vial was observable at this stage. The liquid phase was discarded, and 500 µl of 70 % ethanol was added to the vial. The samples were centrifuged at 14000 rpm for 30 min at 4 °C. Finally, the vials were left at room temperature for 15–30 min for the transparent DNA sediment at the bottom of the vial to dry. Then, 20 µl of sterile water was added, and the samples were kept at room temperature for 1 h to completely dissolve the DNA in water. The extracted DNA samples were stored at −20 °C until qualitative and quantitative DNA analysis.

### 2.2 Quantification and quality assessment of extracted DNA

**Fig. 1:**
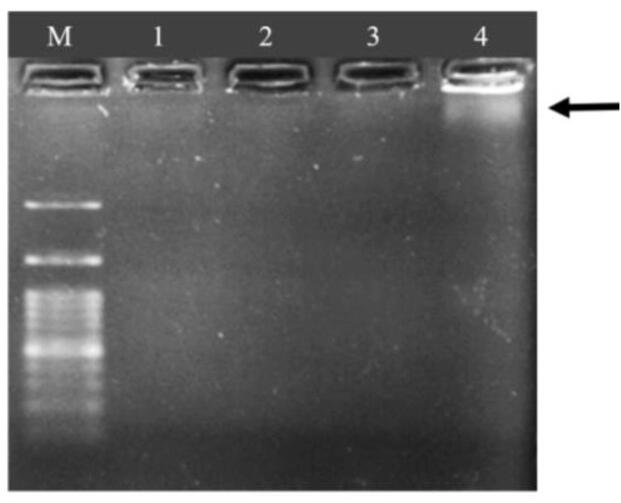
Agarose gel electrophoresis of genomic DNA extracted from crude Soybean Oil. Well 1: DNA extraction using the CTAB method, wells 2 and 3: DNA extracted using the MBST kit, well 4: DNA extracted using the manual hexane-based method, M: Molecular marker.

Before conducting the PCR reaction on the extracted DNA from crude and refined soybean, corn, and canola oils, the quality of the extracted DNA was assessed using agarose gel electrophoresis. The results are presented in Fig. 1.

To quantify the amount of extracted DNA from oil, a NanoDrop device (BioTek Epoch, USA) was utilized. Using this instrument, DNA absorbance at 260 nm (nucleic acid absorption wavelength) and 280 nm (protein absorption wavelength) was measured, and ultimately, the A_260_/A_280_ absorption ratio, which is an indicator of DNA purity, was obtained.

### 2.3 PCR reaction

To confirm the extracted genomic DNA from oil, the PCR technique was employed using internal control gene primers (endogen) including *Lectin* for soybean, *Zein* for corn, and *HMG* for canola. To investigate the transgene of oils, the *EPSPS* gene,^2^ *NptII* gene,^3^ and *NOS*^4^ terminator were utilized. The sequence of the primers used is provided in Table 1. The materials used in each PCR reaction are listed in Table 2. The PCR program for different primers is shown in Table 3. Finally, the PCR products were analyzed using agarose gel electrophoresis on a 1 % Agarose gel **(**Tables 1, 2, 3 **in Appendix)**.

### 2.4 Protein extraction

To assess the protein content in the oil, a PBS buffer was used. The PBS buffer was prepared as follows: 137 mM NaCl, 10 mM KH_2_PO_4_, 1.8 mM K_2_HPO_4_, 2.7 mM KCl were dissolved in 1000 ml of water, and the pH was adjusted to 7.4. Subsequently, 1 ml of oil was poured into a vial, and 200 µl of PBS buffer was added. The mixture was vortexed for 5 min and then placed in a shaker for approximately 1.5 h. Afterward it was centrifuged under the conditions of 10 min, 4 °C, and 12000 rpm. The lower phase containing proteins was transferred to a new vial and used for the quantitative and the quantity and quality of the extracted protein were examined using the Bradford assay and SDS-PAGE^5^ respectively.

#### 2.4.1 Bradford assay

The protein concentration in the extracted sample was determined using the Bradford assay. This colorimetric assay measures the absorbance shift of Coomassie Brilliant Blue G-250 dye in response to protein binding. A standard curve was generated using known concentrations of a protein standard, and the absorbance of the sample was compared to this curve to determine the protein concentration (Bradford, 1976).

#### 2.4.2 SDS-PAGE

The specimens underwent separation 12 % sodium dodecyl sulfate polyacrylamide gel electrophoresis (Simpson, 2006). Subsequently, the gel was dyed with 0.1 % Coomassie brilliant blue G-250 at 45 % methanol and 10 % acetic acid glacial for a minimum of 3 h. Destained was carried out using a solution of 45 % methanol and 10 % glacial acetic acid until the desired background was achieved.

## 3 Results and discussion

### 3.1 Quantitative and qualitative assessment of DNA extracted from oil

The extraction of DNA from oil using the MBST kit was employed to quantitatively measure and assess the purity of the DNA present in the samples. The amount of DNA extracted from soybean, corn, and canola oil samples was measured using the NanoDrop instrument (Table 4) **(**Table 4 **in Appendix)**. In samples tested using this method, the DNA concentration was 113.250–309.929 ng/ml (% 0.125–0.344 × 10^−4^). It should be noted that DNA concentration does not equal GMO content. This concentration is significantly lower than the threshold for labeling GM food products in the strict standards of the European Union and some countries, which is 0.9 % (Avsar et al., 2020, Kaygusuz, 2019, Gao et al., 2018, Coello et al., 2017, Qian et al., 2018, Yang et al., 2005, Pauli et al., 1998, Schmidt et al., 2008, Coello et al., 2017, Iioh et al., 2018, Sepahian et al., 2019, Munteanu et al., 2020).

DNA extraction was successful using both methods, and a satisfactory amount of DNA was extracted from oil samples (Table 4) **(**Table 4 **in Appendix)**. To assess the quality of the extracted DNA, the A_260_/A_280_ absorption ratio can be employed. The A_260_/A_280_ ratio provides an estimate of DNA purity by considering contaminants that absorb UV light, such as proteins. High-quality DNA typically has an A_260_/A_280_ ratio between 1.7 and 2. Strong absorption at A_280_ leads to a decrease in the A_260_/A_280_ ratio, indicating the presence of contaminants like proteins (Olson and Morrow, 2012). The A_280_/A_260_ absorption ratio for DNA extracted from crude and refined soybean, corn, and canola oil samples using the MBST kit ranged from 1.55 to 1.92. For manual hexane-based extraction, the A_280_/A_260_ ratio fell within the range of 1.73 to 1.92. The purity of extracted DNA in terms of protein with both extraction methods was evaluated for PCR to be suitable. In the extraction of genomic DNA from oil using the CTAB method, acceptable DNA for use in PCR was not obtained; therefore, data related to extraction with this method was not presented.

Furthermore, the results indicated that the DNA extraction method using the MBST kit yields an acceptable amount of DNA for GMO analysis, but this method failed to produce successful PCR reactions due to the presence of PCR inhibitors. PCR inhibitors are substances that either directly interact with DNA or block the activity of the Taq polymerase enzyme, hindering the replication of DNA in the PCR reaction (Bessetti, 2007). The likely reason for this may be the presence of Guanidine in the DNA extracted using BST kit. Guanidine is a strong PCR inhibitor that hinders the binding of DNA to Taq polymerase, consequently inhibiting PCR (Hale et al., 1996). Most DNA extraction kits utilize guanidinium lysis as a primary step, followed by the capture of nucleic acid using silica (Bosch et al., 2011). Therefore, for the investigation of the genetic modification in soybean, corn, and canola oils, DNA extracted through the manual hexane-based method was utilized.

Agarose gel electrophoresis was employed to assess the quality of the extracted DNA. The results demonstrated the presence of DNA in crude soybean oil extracted using the manual hexane-based method (Fig. 1, well 4), while no DNA was observed in the agarose gel corresponding to DNA extracted using the MBST and CTAB methods (Fig. 1). Based on the quantitative analysis of the extracted DNA from oil using the Nano Drop, the calculated percentage of DNA in 1 ml of oil for all three methods was very low, almost close to zero (Table 4) **(**Table 4 **in Appendix)**.

One of the common challenges in DNA extraction from oil, as almost all previous studies have indicated, is the low quantity and impurity of DNA in oil samples. This is a common method where crude oil, before being introduced to the market for human consumption, undergoes purification to eliminate undesirable odor and color (Gryson et al., 2004a, Gryson et al., 2004b). The extraction of oil and subsequent refining processes contribute to the degradation of genomic DNA in the purified oil, rendering it odorless (Gryson et al., 2004a, Gryson et al., 2004b). Manual hexane DNA extraction from crude soybean oil showed good results, with observable DNA bands on agarose gel electrophoresis (Fig. 1). The observed band was a result of the pellet fraction derived from 12 ml of crude oil containing soy cells, demonstrating successful DNA extraction.

### 3.2 The investigation of the presence of the *Lectin* gene in crude and refined soybean oil

**Fig. 2:**
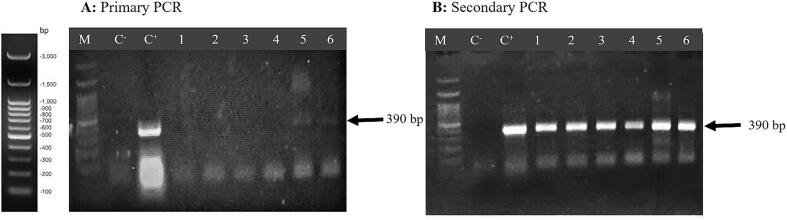
Agarose gel electrophoresis of PCR products amplified from DNA extracted from crude and refined soybean oil using the manual hexane-based method. A: Primary PCR, B: Secondary PCR, C^−^: Negative control (water), C^+^: Positive control (soybean leaf DNA), well 1: pellet phase crude soybean, well 2: liquid phase crude soybean, Well 3: pellet phase refined soybean, well 4: liquid phase refined soybean, wells 5 and 6: crude and refined soybean oil were directly added as a template to the PCR reaction, respectively, M: Molecular marker (bp 100).

After manual hexane DNA extraction and quantification and quality assessment using Nano Drop and agarose gel, a PCR reaction was performed on the genomic DNA extracted from crude soybean oil. This was accomplished using specific primers for the *Lectin* gene, resulting in the amplification of a fragment approximately 390 bp in size in both pellet and liquid samples of crude soybean oil after two rounds of PCR. Consequently, the presence of the *Lectin* gene in both crude and refined soybean oil was confirmed. This confirmed, firstly, the existence of DNA in both crude and refined soybean oil, and secondly, that this DNA corresponded to the soybean genome. The results of agarose gel electrophoresis for the primary and secondary PCR products, derived from the amplification of existing DNA extracted from crude and refined soybean oil, are illustrated in Fig. 2A and 2B, respectively. In this study, in cases where PCR products obtained from DNA extraction from oil, refined oil, or crude oil were used as templates, and no amplification was achieved, the products of these PCRs were utilized as templates for the subsequent PCR, referred to as secondary PCR.

As observed in Fig. 2A and 2B, the DNA extracted from crude and refined soybean oil using the manual hexane method had minimal amplification capability in the primary PCR. The band corresponding to the *Lectin* gene on the agarose gel electrophoresis was barely visible in the primary PCR (Fig. 2A). However, when the products of the primary PCR were used as templates (secondary PCR), the amplified DNA fragment (bp390) became clearly visible as distinct bands (Fig. 2B).

An interesting point regarding soybean oil is that when crude and refined soybean oil were directly added as a template to the PCR reaction, the amplification of the 390 bp band confirming the presence of DNA in these oils was evident (Fig. 2A, wells 5 and 6). This indicates that there are inhibitors present in the DNA extracted from crude and refined soybean oil using the manual hexane method. With the secondary PCR, the inhibitory effects decreased, and DNA amplification increased. It appears that during the oil extraction and purification process of soybean oil, cells may be present in the oil. Therefore, when crude and refined soybean oil was used as a template in the PCR reaction, the presence of genomic DNA in the oil matrix led to the amplification of *Lectin* gene DNA in PCR (Fig. 2).

### 3.3 The investigation of the presence of the *Zein* gene in crude and refined corn oil

**Fig. 3:**
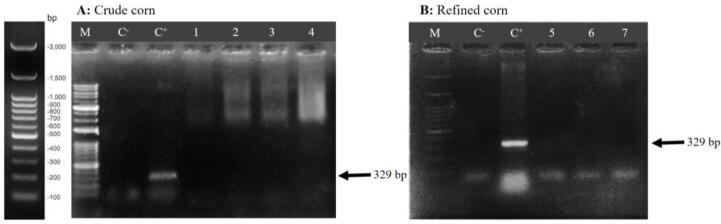
Agarose gel electrophoresis of PCR products amplified from DNA extracted from crude and refined corn oil using the manual hexane-based method. C^−^: Negative control (water), C^+^: Positive control (corn leaf DNA), wells 1 to 4: 0.5, 1.0, 1.5, and 2.0 µl of DNA extracted from pellet phase crude corn as templates in PCR reaction, respectively, well 5: pellet phase refined corn, well 6: liquid phase refined corn, well 7: refined corn oil were directly added as a template to the PCR reaction, M: Molecular marker (100 bp).

DNA extraction from the pellet fraction of 12 ml crude corn oil was carried out using the manual hexane DNA extraction method. PCR was performed using primers specific to the *Zein* gene of corn. For this purpose, different amounts of DNA extracted from the plate fraction (0.5, 1, 1.5, and 2 µl) were used as templates in the PCR to obtain the optimal concentration that would provide better amplification and consequently produce clear bands. The agarose gel electrophoresis results of the PCR products of these samples showed that the amounts of 1, 1.5, and 2 µl of DNA extracted by the manual hexane method from the pellet fraction of crude corn oil demonstrated amplification (Fig. 3A), confirming the presence of DNA in crude corn oil.

In the case of refined corn oil, no amplification was observed in PCR. Fig. 3B indicates that none of the DNA samples extracted from the pellet and liquid fractions of refined corn oil, and even the refined corn oil directly used in the PCR reaction, showed the ability to amplify with the *Zein* primers of corn.

### 3.4 The investigation of the presence of the *HGM* gene in crude and refined canola oil

**Fig. 4:**
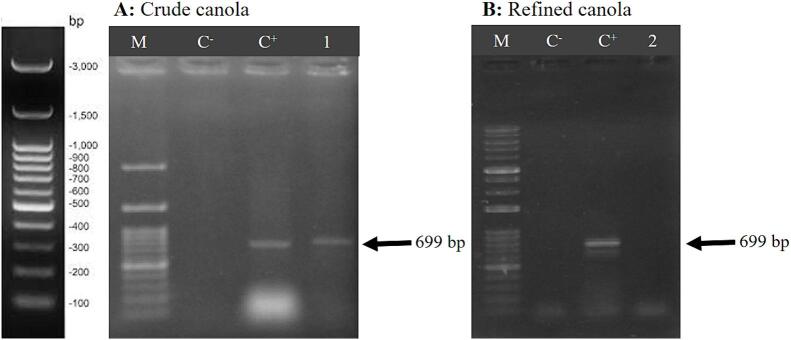
Agarose gel electrophoresis of PCR products amplified from DNA extracted from crude and refined canola oil using the manual hexane-based method. C^−^: Negative control (water), C^+^: Positive control (canola leaf DNA), well 1: pellet phase crude canola, well 2: pellet phase refined canola M: Molecular marker (100 bp).

The presence of DNA in canola oil was confirmed using the PCR technique with primers specific to canola gene *HGM*. The PCR with DNA extracted manually hexane method was successful. The presence of a 699 bp band confirmed the existence of DNA in canola oil, and this DNA was related to the canola genome (Fig. 4A). The results of agarose gel electrophoresis of the PCR product obtained from manually hexane-extracted DNA from crude and refined canola oil are shown in Fig. 4A and 4B, respectively.

The results demonstrated that the extracted DNA from the pellet section of crude canola oil had the ability to amplify with primers specific to the canola gene *HGM* in the primary PCR. Thus, the presence of the genome in crude canola oil was confirmed (Fig. 4A). The agarose gel electrophoresis results of the PCR products obtained from refined canola oil, DNA extracted using the manual hexane method, are shown in Fig. 4B. Regarding refined canola oil, the *HGM* gene showed no amplification in both the primary and secondary PCR.

Understanding and managing the impact of PCR inhibitors on the quality and reliability of PCR is crucial for ensuring accurate results. The absence of DNA detection and amplification in refined corn oil (Fig. 3B) and refined canola oil (Fig. 4B) does not indicate a lack of DNA in these oils. Quantitative analysis of DNA extracted from refined corn oil and refined canola oil confirmed the presence of genomic DNA in the oils. Therefore, DNA is indeed present in corn oil and canola oil, but due to the presence of PCR (Taq polymerase enzyme) inhibitors, it has not been able to amplification. The major challenge of DNA extraction from vegetable oil is the high amount of PCR inhibitors in vegetable oils, such as polysaccharide, phenol, and other compounds that are not completely removed during DNA extraction protocols and these substances are PCR inhibitors (Costa et al., 2010, Costa et al., 2010). In addition to various salts (e.g., sodium chloride or potassium chloride), the presence of detergents or organic molecules (ethylene-diamine-tetra-acetic acid, sarkosyl, ethanol, isopropyl alcohol or phenol) in the DNA extraction protocol can inhibit PCR at specific concentrations (Piarulli et al., 2019). Therefore, caution should be taken, and if these substances are used in the DNA extraction protocol, they should be removed through repeated washes.

The challenge of extracting the genome from vegetable oils, compounded by the low quantity of genome present, is further complicated by the risk of contamination from the genomes of other organisms. To validate the genome extracted from the oil, the PCR technique was employed, utilizing specific primers for key genes: *Lectin* for confirming the soybean genome, *Zein* for confirming the corn genome, and *HMG* for confirming the canola genome. These genes were designated as internal control genes or reference genes. This approach is crucial for ensuring the accuracy of the test results. Internal control genes play a pivotal role in validating the genome by verifying the proper functioning of the PCR reaction and the reliability of the results. Moreover, using these control genes helps guarantee that the DNA extracted from the oil remains uncontaminated by other sources, thereby preserving the integrity of the results (De la Torre et al., 2004).

### 3.5 Reviewing the genetically modified status of Soybean, Corn, and canola oils

**Fig. 5:**
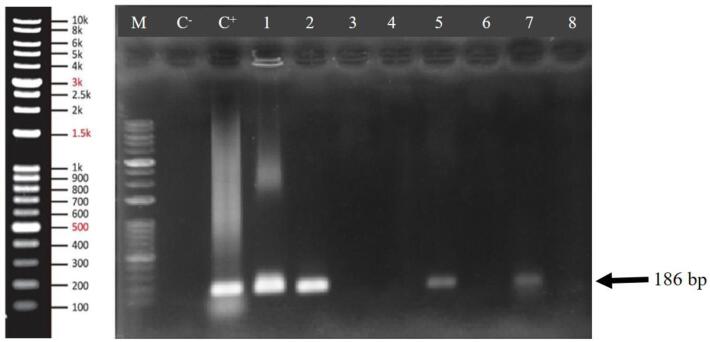
Agarose gel electrophoresis of PCR products amplified from DNA extracted from crude and refined soybean, corn, and canola oils using NOS primers. C^−^: Negative control (water), C^+^: Positive control (plasmid containing NOS sequence), well 1: pellet phase crude soybean, well 2: liquid phase crude soybean, wells 3: pellet phase refined soybean, well 4: liquid phase refined soybean, well 5: pellet phase crude corn, well 6: pellet phase refined corn, well 7: pellet phase crude canola, well 8: pellet phase refined canola, M: Molecular marker.

After confirming the presence of DNA in crude and refined soybean, corn, and canola oils and verifying their genomes using internal control gene primers, to assess their transgenic, the most common transgenic primers such as NptII, EPSPS, and NOS were employed. The PCR results using the NOS primer in Fig. 5 indicate that soybean, corn, and canola oils are transgenic. The presence of the 186 bp band confirmed that these oils were indeed transgenic.

NptII primers, associated with resistance to the kanamycin antibiotic in corn, canola, and soybean oils, and EPSPS primers were employed to assess resistance to Roundup herbicide in soybean oil for confirming transgenicity. PCR results did not yield any amplification with these primers (results not shown). The probable reason for this could be the absence of these genes in the genome. Another possibility for the non-amplification of these genes in PCR may be related to the gene sequence. If the codon preference is done for these genes, the DNA sequence changes without the protein sequence changing. In such cases, the primer design from the gene to confirm transgenicity will be unsuccessful. In this situation, using degenerate primers is a good solution. It was also checked oils were transgenic with the CaMV35S primer, and no amplification was observed.

The results of this research show that DNA fragments can be identified and measured in some oil samples, and this result is in agreement with the research of some researchers (Hellebrand et al., 1998; Zhang et al., 2007; Schmidt et al., 2008, Costa et al., 2010, Costa et al., 2010, Luna et al., 2013, Nikolić et al., 2014, Shayan et al., 2017, Iioh et al., 2018, Sepahian et al., 2019, Piarulli et al., 2019, Fröder et al., 2020, Xia et al., 2021). Also, the investigation of some researchers indicates that due to the destruction of DNA in the oil refining process, it is not possible to identify GMO products in the oil, and there is no need for labeling (Pauli et al., 1998, Gryson et al., 2002, Gryson et al., 2004a, Gryson et al., 2004b, Costa et al., 2010, Costa et al., 2010, Jinxia et al., 2011).

In this research, significant efforts were made to optimize DNA extraction methods from oil to not only increase the DNA amount but also ensure its purity for PCR applications. One optimization step involved increasing the tested oil sample volume from 1 ml to 12 ml (for corn). With the increase in sample volume, a higher amount of DNA was ultimately obtained. Another optimization measure was the use of proteinase K to eliminate substantial protein content present in the oil, resulting in extracted DNA free from proteins. Additionally, various volumes of the extracted DNA (0.5, 1, 1.5, and 2 µl) were used as templates in the PCR reaction. This approach was chosen because the concentrations of inhibitors in the extracted DNA were uncertain, and using different volumes in the PCR reaction helps determine the most suitable amount for amplification. This can be a strategy to achieve sharp bands and high amplification in PCR.

Quantitative data indicate that manual hexane-based DNA extraction from refined soybean, corn, and canola oils results in a lower amount of DNA compared to their crude (unrefined) counterparts. Therefore, it can be stated that the purification process is effective in reducing the DNA content in the refined (odorless) oils. Our results align with other conducted research, including studies by Gryson et al. (2004), Gryson et al. (2002), and Pauli et al. (1998). According to conducted studies, all purification stages, especially thermal processes, the use of activated clay, and induced pH changes (using organic acids and sodium hydroxide), may have an impact on the quality and quantity of the remaining DNA in fully purified oil, leading to a reduction in the DNA content of the oil.

### 3.6 Investigating the presence of protein in oil

**Fig. 6:**
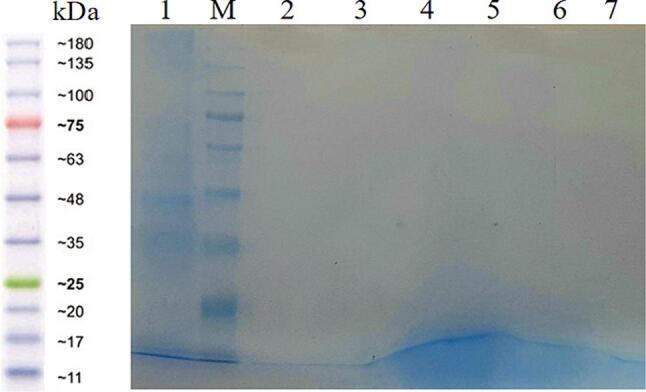
Quality assessment of extracted protein from oils. Well 1: protein extracted from leaf, well 2: refined Soybean, well 3: refined Canola, well 4: refined Corn, well 5: Crude Soybean, well 6: Crude Canola, well 7: Crude Corn, M: Molecular weight marker.

The results of protein quantification using the Bradford method indicated the presence of protein in crude and refined soybean, corn, and canola oils (Table 5) **(**Table 5 **in Appendix)**, while SDS-PAGE gel results of proteins extracted from the oils did not exhibit any protein bands (Fig. 6). The possible reason for the inconsistency in observations regarding the quantity and quality of extracted protein from oils could be attributed to protein molecules being destroyed in the oil.

The Bradford reagent, composed of Coomassie Brilliant Blue G-250, methanol, and orthophosphoric acid, is commonly used for protein quantification. The effectiveness of Bradford assay relies on the interaction with Coomassie dye. Its ability to react with a specific set of amino acids (Arg, His, Lys, Phe, Tyr, and Trp) makes it a suitable chemical method for protein analysis. Coomassie Brilliant Blue binds to proteins both through hydrophobic reactions (via Phe, Trp, etc.) and electrostatic attraction between the sulfonic group and the guanidine group with a positive charge on Arg (secondarily with Lys, His) in proteins. Therefore, evidence suggests that this reagent can only bind to protein molecules (Brunelle et al., 2017, Grintzalis et al., 2006). Estimating protein amounts using Bradford may be a result of interactions between Coomassie Blue and broken-down protein molecules in crude and refined soybean, corn, and canola oils. While these small fragmented proteins easily migrate out of the SDS-PAGE gel, hence no protein bands are observed in samples extracted from the oils (Fig. 6). It is also possible that the levels of intact proteins in the oil were very low, making them undetectable using SDS-PAGE gel.

Determining the protein content of oils is of vital importance because proteins are potentially responsible for any allergic properties. Allergenic proteins are relatively small and resistant to heat, acid, and gastric enzyme degradation (van Ree, 2002). Therefore, quantifying protein content is crucial for assessing potential risks. Available data indicates that the protein content in crude oil is in the range of 100–300 µg, and oil refining leads to levels about 100 times lower. Crevel and colleagues concluded that peanut oil and, by extrapolation, other edible vegetable oils pose no risk of triggering allergic reactions in the majority of susceptible individuals (Crevel et al., 2000).

Genetically modified (GM) food technology presents a promising solution to contemporary and future challenges in medicine and food. Nonetheless, substantial apprehension exists regarding safety of genetically modified foods for human consumption, often fueled by criticisms lacking sufficient evidence. Some countries have refrained from using genetically modified foods for famine relief due to perceived health risks. Key concerns involve potential allergenicity and toxicity, even though GM foods undergo rigorous testing before receiving market approval (Lee et al., 2017).

The belief that allergic reactions may result from the consumption of genetically modified oils has yet to be proven. The World Health Organization has declared that no allergic reactions related to the consumption of genetically modified products have been identified so far (World Health Organization, 2016). Despite this statement, genetically modified foods available for public consumption have undergone thorough risk assessments, including sensitivity tests. Foods derived from genetically modified technology have been consumed by millions worldwide without any reported adverse effects (Bradford et al., 2005). One common strategy to address concerns is the implementation of mandatory labeling for genetically modified products.

In the year 2000, Grace Booth in the United States experienced anaphylaxis after consuming corn tacos. Subsequently, it was revealed that some taco shells contained a pesticide protein called Cry9C, originating from *Bacillus thuringiensis*. Cry9C was incorporated into genetically modified corn to combat certain predatory insects and was initially approved only for animal feed. However, due to cross-pollination, it entered the human food chain when GM crops were planted in close proximity to conventional crops. Although other causes of Booth's anaphylaxis could not be identified, the Cry9C protein was assumed to be the culprit. The Centers for Disease Control and Prevention in the United States never directly linked Cry9C and the allergy development. However, this incident contributed to the public and media perception that genetically modified foods might lead to new allergies (Lee et al., 2017).

Opponents of genetically modified (GM) technology have proposed a connection between genetically modified foods and the notable rise in food allergies in the United States, particularly among children. Nevertheless, widely recognized food allergens like eggs, dairy, shellfish, tree nuts, and peanuts, known for triggering allergies, cannot be directly tied to GM technology. Consequently, the increase in the prevalence of these common food allergies cannot be directly attributed to GM technology (Lee et al., 2017).

## 4 Conclusion

The extraction of genomic DNA from oil poses a significant challenge due to the minimal presence of genomic material in the oil matrix. The manually performed hexane method yields DNA quantities with sufficient quality for investigating the genetic makeup of edible oils. In this study, the manual hexane extraction method was evaluated as an ideal approach for extracting DNA from edible oils. Optimization steps included increasing the oil sample quantity, utilizing proteinase K to eliminate excessive protein content in the oil, and adjusting the template amount in the PCR reaction to enhance band sharpness. Quantitative data from this research indicated that manual hexane extraction of DNA from soybean, corn, and canola oils resulted in lower DNA amounts in refined oils compared to their crude counterparts. Nevertheless, the DNA concentrations in all three oils tested were well below the threshold for labeling GM food products set by the stringent standards of the European Union and some countries, i.e., 0.9 %. Additionally, the protein content in crude and refined soybean, corn, and canola oils was found to be extremely low, to the extent that no protein bands were observed in SDS-PAGE gel.

## Compliance with ethical standards

### Funding

This research did not receive any specific grant from funding agencies in the public, commercial, or not-for-profit sectors.

## Ethical approval

This article does not contain any studies with human participants or animals performed by any of the authors.

## Informed consent

Informed consent was obtained from all individual participants included in the study.

## CRediT authorship contribution statement

**Melika Vahdani:** Visualization, Software, Methodology, Formal analysis. **Mohammad Ali Sahari:** Writing – review & editing, Writing – original draft, Visualization, Validation, Supervision, Resources, Project administration, Investigation, Data curation. **Mehrnaz Tanavar:** Writing – original draft, Validation, Project administration, Investigation, Formal analysis.

## Declaration of competing interest

The authors declare that they have no known competing financial interests or personal relationships that could have appeared to influence the work reported in this paper.

## Figures and Tables

**Table 1:** Specifications of primers used in PCR.

Reference	Annealing (˚C)	Length	Sequence	Primer name
Present study	55.2	390 bp	GACACTAAGCCACAAACACATGC	Lectin F
			AGACCAAGAAAGCACGTCATG	Lectin R
ISO 21569, Amendment 1, 2013-04-01	60	329 bp	TGCTTGCATTGTTCGCTCTCCTAG	Zein F
			GTCGCAGTGACATTGTGGCAT	Zein R
Present study	57.1	669 bp	GGCACGAGCTCTCTCCTACC	HMG-F
PanL et al., 2007			GCAACCAACAGGCACCATC	HMG-R
Collection of official methods under article 35 of the German Federal Foodstuffs Act. 1998	58	172 bp	TGATGTGATATCTCCACTGACG	EPSPS F
			TGTATCCCTTGAGCCATGTTGT	EPSPS R
Ping-Ping et al., 2008	58	364 bp	CCTTGAGCCTGGCGAACA	NptII F
			GGTGCCCTGAATGAACTGC	NptII R
ISO 21569, Amendment 1, 2013-04-01; James et al., 2003	56.1	186 bp	GAATCCTGTTGCCGGTCTTG	NOS F
Present study			GATAATTTATCCTAGTTTGCGCGCT	NOS R

**Table 2:** Materials used in each PCR reaction.

Reagent	Concentration of stock solutions	Final concentration	Volume
PCR Buffer	10 X	1 X	2 µl
dNTP’s	10 mM	0.1 mM	0.6 µl
MgCl_2_	50 mM	1.5 mM	0.2 µl
Forward primer	10 mM	0.2 mM	0.4 µl
Reverse primer	10 mM	0.2 mM	0.4 µl
Taq DNA polymerase	5 Units/µl	0.3 U	0.4 µl
Template DNA	Variable	50 – 500 ng	Variable
Sterile H_2_O	–	–	Up to 20 µl
Total volume	–	–	20 µl

**Table 3:** PCR program for primers targeting different genes.

	*Lectin* gene	*Zein* gene	*HMG* gene	*NOS*
Initial denaturation	94 ˚C for 5 min	94 ˚C for 5 min	94 ˚C for 5 min	94 ˚C for 5 min
Denaturation	94 ˚C for 45 s	94 ˚C for 30 s	94 ˚C for 45 s	94 ˚C for 30 s
Annealing	55.2 ˚C for 30 s	60 ˚C for 30 s	57.1 ˚C for 30 s	56.1 ˚C for 30 s
Extension	72 ˚C for 23 s	72 ˚C for 20 s	72 ˚C for 30 s	72 ˚C for 20 s
Final extension	72 ˚C for 5 min	72 ˚C for 5 min	72 ˚C for 5 min	72 ˚C for 5 min
Cycle	35	35	35	35

**Table 4:** Concentration and purity of extracted DNA from oils. The data represented the mean ± standard error (±SE) of three independent replicates.

Oil type	DNA extraction method	DNA Concentration (ng/mL)	Purity (A_260_/A_280_)	DNA concentration (%)
Crud soybean	MBST kit	309.92 ± 51.71	1.78 ± 0.091	0.0000344 ± 0.0000057
Refined soybean	MBST kit	263.43 ± 113.76	1.91 ± 0.096	0.0000292 ± 0.0000126
Crud corn	MBST kit	254.36 ± 68.14	1.79 ± 0.100	0.0000282 ± 0.0000075
Refined corn	MBST kit	248.19 ± 105.42	1.92 ± 0.206	0.0000275 ± 0.0000117
Crud canola	MBST kit	113.25 ± 15.68	1.55 ± 0.313	0.0000125 ± 0.0000017
Refined canola	MBST kit	159.25 ± 42.28	1.86 ± 0.210	0.0000176 ± 0.0000046
Crud soybean	Manual hexane-based	67.07 ± 4.30	1.73 ± 0.07	0.0000074 ± 0.0000009
Refined soybean	Manual hexane-based	29.125 ± 3.77	1.9 ± 0.14	0.0000032 ± 0.0000008
Crud corn	Manual hexane-based	70.35 ± 8.704	1.92 ± 0.07	0.0000078 ± 0.0000019
Refined corn	Manual hexane-based	48.32 ± 0.10	1.91 ± 0.12	0.0000026 ± 0.0000037
Crud canola	Manual hexane-based	325.155 ± 88.42	1.75 ± 0.02	0.0000361 ± 0.0000196
Refined canola	Manual hexane-based	72.135 ± 1.87	1.85 ± 0.13	0.0000080 ± 0.0000004

**Table 5:** Quantitative protein amounts extracted from 1 ml of crude and refined soybean, corn, and canola oils using the Bradford method. The data represented the mean ± standard error (±SE) of three independent replicates.

Oil type	Protein concentration (mg/mL)
Crud soybean	4.4979 ± 0.01956
Refined soybean	5.333 ± 0.0265
Crud corn	5.2816 ± 0.03648
Refined corn	5.4464 ± 0.01891
Crud canola	4.3637 ± 0.02477
Refined canola	4.4531 ± 0.0224

## Data Availability

The authors do not have permission to share data.

## Footnotes

1 International Service for the Acquisition of Agri-biotech Applications.

2 glyphosate-resistant gene encoding 5-enolpyruvylshikimate-3-phosphate synthase enzyme.

3 antibiotic kanamycin-resistant gene.

4 Nopaline synthase terminator.

5 Sodium Dodecyl Sulfate-Polyacrylamide Gel Electrophoresis.
